# Mpox Cases in Serbia, 2022

**DOI:** 10.3390/idr17010009

**Published:** 2025-01-27

**Authors:** Petar Đurić, Verica Jovanović, Mitra B. Drakulović, Dragana Plavša, Jovan Malinić, Aleksandar Medarević, Jelena Protić, Nana Mebonia

**Affiliations:** 1 National Public Health Institute “Dr. Milan Jovanović Batut”, 11000 Belgrade, Serbia; 2 Mediterranean and Black Sea Program in Intervention Epidemiology Training (MediPIET), European Center for Disease Prevention and Control (ECDC), 16973 Stockholm, Sweden; 3 Clinic for Infectious and Tropical Diseases, University Clinical Center of Serbia, 11000 Belgrade, Serbia; 4 The Institute of Virology, Vaccines, and Sera “Torlak”, 11000 Belgrade, Serbia

**Keywords:** mpox, outbreak, men who have sex with men, Serbia, sexually transmitted infections

## Abstract

Background: On 23 July 2022, the World Health Organization (WHO) declared the mpox multi-country outbreak as a Public Health Emergency of International Concern. This study aimed to identify the epidemiological and clinical characteristics of confirmed mpox cases reported in Serbia in 2022. Methods: The mpox WHO case definition was used. Incidence rates (IRs) and incidence rate ratios (IRRs) by age groups and nomenclature of territorial units for statistics level 3 (NUTS-3) with 95% confidence intervals (CIs) were calculated. Results: Between June and October 2022, 43 laboratory-confirmed cases were reported. All were unvaccinated males, with the mean age of 34 (±7.4) years. Out of the total, 72.1% cases were men who have sex with men (MSM), who reported sexual intercourse either with multiple or unknown partners (*p* < 0.01). Fifteen cases (34.9%) lived with HIV, mostly in the 30–39 age group (*p* = 0.023). People living in Belgrade City NUTS-3 were six times more likely to become infected compared to South Backa citizens (IRR: 6.03, 95% CI: 1.47–25.53). Conclusions: In Serbia, mpox mainly affected MSM aged 30–39 and living in urban areas. Health promotion and vaccine implementation should be prioritized in populations with a higher risk.

## 1. Introduction

Mpox is an infectious disease caused by the human *monkeypox virus* (MPXV), a double-stranded DNA virus belonging to the *Orthopoxvirus genus* and *Poxviridae* family [1,2]. A total of 28 Poxviridae family genera are known, but only four of them cause diseases in humans: Orthopoxviridae, Parapoxviridae, Yatapoxviridae, and Molluscipoxviridae. The mpox virus, vaccinia virus, and smallpox virus (Variola vera) belong to the Orthopoxviridae genus and are spread by direct contact and typically cause papular, pustular, or crusted rashes [1,2]. Unlike MPXV, a smallpox virus causes residual skin scars and disease with higher lethality [3]. The Molluscipoxviridae genus, the molluscum contagiosum virus, has the same transmission path and causes translucent, umbilicated skin papules without systemic symptoms [1,4].

Rodents and primates are most likely reservoirs of MPXV. It was isolated for the first time in 1958, in Denmark in *Cynomolgus* monkeys. The first known human case occurred in 1970 in the Democratic Republic of the Congo [5]. Thereafter, mpox circulated, causing zoonotic disease outbreaks in endemic areas, such as Central and Western Africa, until 2003, when an outbreak was reported from the United States of America (USA) in humans, where the main transmission route was close contact between pets and rodents imported from Ghana [6]. Other examples of human mpox outbreaks in the United Kingdom, Israel, Singapore, and the USA were associated with recent travel to endemic areas, and these cases were considered imported [7,8,9,10]. Furthermore, in May 2022, several non-endemic countries started to report mpox cases without a travel history before disease onset or contact with infected animal reservoirs [11,12]. On 23 July 2022, the World Health Organization (WHO) declared the multi-country outbreak of mpox as a Public Health Emergency of International Concern. From 1 January 2022 up to 30 November 2023, more than 90,000 mpox cases were confirmed, and 171 cases of death were reported to the WHO from 116 countries [13]. The most affected country was the USA, with 33% of the total number of confirmed cases, followed by Brazil (11.8%) and Spain (8.3%) [13]. The majority of cases occurred among males, and a high proportion of them were men who have sex with men (MSM) and people living with human immunodeficiency viral (HIV) infection. The main transmission routes recognized among the confirmed cases were close physical and sexual contact between susceptible and infected persons [14]. People vaccinated against smallpox are partially protected against the severe mpox disease due to the cross-reaction of neutralizing anti-smallpox antibodies to monkeypox virus antigens [15]. In July 2022, previously approved for immunization against smallpox in 2013, the third-generation smallpox vaccine has been approved in the EU as an immunization agent against mpox [16].

In late May 2022, Serbia established a nationwide enhanced mpox surveillance system to detect mpox cases and control further transmission in the community. At the same time, mpox disease became notifiable, according to the Serbian Law on the Protection of Population from Infectious Disease [17] and Commission Implementing Decision (European Union), 2018/945 [18]. On 17 June 2022, the first mpox case was identified. Thereafter, the number of mpox cases gradually increased, reaching a total of 43 confirmed cases throughout Serbia in less than four months.

This study aimed to identify the epidemiological and clinical characteristics of confirmed mpox cases in Serbia during the disease outbreak in 2022.

## 2. Materials and Methods

### 2.1. Data Collection

An enhanced, case-based surveillance system was carried out in Serbian healthcare facilities (HFs) across 25 nomenclature of territorial units for statistics level 3 (NUTS-3) of the country. The WHO case definition for the mpox was used [19]. Suspected mpox cases were medically examined, and clinical specimens including oropharyngeal and skin-lesions swabs were taken and sent for laboratory analyses to the reference laboratory (RL) for rubella, measles, varicella, and other febrile rash illnesses at the Institute of Virology, Vaccines, and Sera “Torlak” in Belgrade, where real-time polymerase chain reaction (RT-PCR) was performed. Furthermore, the RL forwarded the laboratory results to HFs, the Centers for Disease Control (CDCs) of NUTS-3 level Institutes of Public Health (IPH-3), and the CDC of the National Institute of Public Health (NIPH).

In addition, cases with positive RT-PCR results were notified by HFs to competent CDCs of IPH-3s, and consequently, their epidemiologists carried out case investigations using a standardized case investigation form (CIF) and contact tracing (using a contact tracing form). Close contacts of cases, such as household members, school peers, work colleagues, sexual contacts, contacts within HFs, and mass gatherings, were identified and traced by CDC epidemiologists and sanitary engineers over 21 days. The CIFs of all confirmed cases were forwarded to the National CDC, Coordinating Center for Surveillance established in the NIPH. The CDC of the NPHI notified confirmed cases through the National Informatics System for Public Health Services and reported these to the Ministry of Health of Serbia, the European Surveillance System (TESSy), the European Center for Disease Prevention and Control, and the WHO.

Cases were classified by their HIV status, based on their reporting during the case investigation process, as follows: HIV-positive, HIV-negative, and unknown HIV status. Those marked as “negative” declared that they did not have any of the conditions such as STIs, HIV infection, pregnancy, or other diseases. We did not perform testing for HIV infection among them during the case investigation process. Considering this, we marked them as unknown.

### 2.2. Data Analysis

We conducted a descriptive observational study of confirmed and notified mpox cases from Serbian’s mpox surveillance system. We obtained anonymized individual data including socio-demographic (age, sex, residence by NUTS-3, educational level), epidemiological (day of symptoms onset, mpox and smallpox vaccination status, travel in past 21 days, risky sexual behavior and practices, potential route of transmission, contact with probable or confirmed case, HIV coinfection, previous sexually transmitted infections (STI) history), and clinical data (presence of fever, lymphadenopathy, headache, myalgia, asthenia, and rash morphology and distribution). Male cases who reported sex with men were considered as MSM persons. Based on information from the cases, persons who reported HIV coinfection were classified as persons living with HIV (PLHIV), while those indicating the absence of HIV infection were negative.

Numerical results were reported as medians with interquartile range (IQR), means with standard deviation (SD) or as proportions, and categorical/binary variables using absolute numbers and proportions. The Kolmogorov–Smirnov and Shapiro–Wilk tests were used for normality distribution testing. For group comparison, the Mann–Whitney or Kruskal–Wallis test was performed for numerical data, and Pearson’s Chi-squared test or Fisher’s exact test for categorical/binary data. Incidence rates (IRs) and incidence rate ratios (IRRs) by age groups and geographical areas with 95% confidence intervals (CIs) were calculated. A *p*-value <0.05 was considered to be statistically significant. Statistical analysis was performed using the R statistical software (version 4.4.0). For the geographical visualization of IRs across the NUTS-3 of Serbia, we used the QGIS Geographic Information System, version 3.34.2, Open Source Geospatial Foundation Project.

Swabs of the oropharyngeal region and skin lesions were collected and transported in a commercially available universal transport medium. Nucleic acid extraction was performed using the LightMix Modular Monkeypox Virus Kit (TIB MOLBIOL Sytheselabor GmbH, Berlin, Germany). LightMix tests confirmed the presence of MPXV nucleic acid by 106 bp long fragment amplification of the J2L/J2R gene. Ct ≤ 37 findings were considered positive. A traditional dideoxynucleotide Sanger sequencing of the A56R gene and results interpretation were performed on an Applied Biosystem 3500 Genetic Analyzer (Applied Biosystem, Carlsbad, CA, USA) automatic sequestrator.

## 3. Results

**Figure 1:**
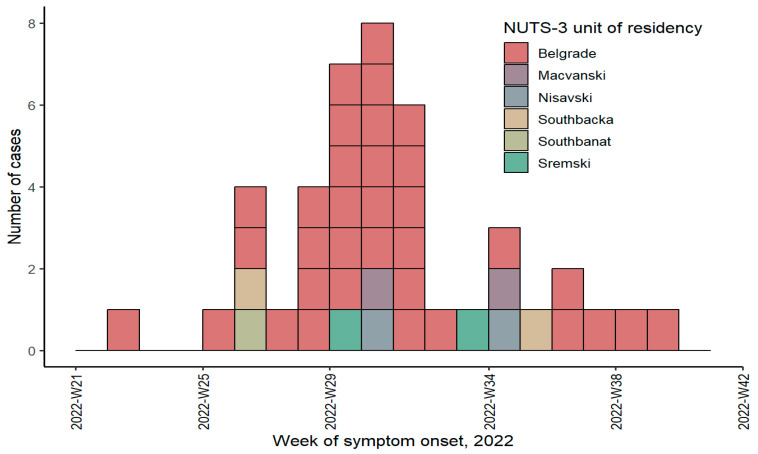
Mpox cases by week of symptoms onset, Serbia, 17 June to 5 October 2022.

Between June and October 2022, 43 laboratory-confirmed mpox cases were reported, according to the national surveillance system (Figure 1).

All cases were adult males. The mean age of cases was 34 (±7.4) years, with the highest proportion belonging to the 30–39 age group (51.2%).

**Figure 2:**
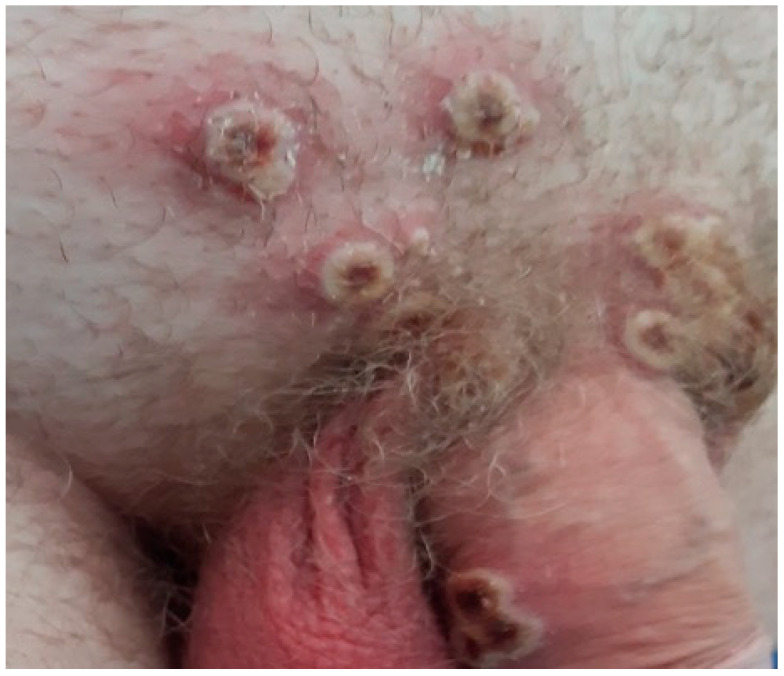
Pustular rash on the pubic and genital area of an mpox case in Serbia, 2022.

Rash occurred in all surveyed cases. The most affected body locations included the anogenital (58.1%, Figure 2), face, head, and neck (27.9%), and trunk (25.6%). Among the cases, skin lesions presented as pustules (93.0%), crusts (69.8%), and vesicles (9.3%) at the moment of the first medical examination. Furthermore, fever was present in 86.0%, followed by lymphadenopathy (44.2%), headache (37.2%), and back pain (34.9%). Three cases (7.0%) required hospitalization in Belgrade City NUTS-3 (two) and South Backa NUTS-3 (one) (Table 1).

Most cases were reported from 28 to 31 calendar weeks, from the middle of July to the beginning of August 2022. The disease onset peaked in the fourth week of July (Figure 1). Mpox cases occurred in six out of twenty-five Serbian NUTS-3 units. The highest number of cases (34, 79.1%), was registered in Belgrade City NUTS-3, followed by South Backa, Macva, Nisava, and Srem NUTS-3 with two cases each (4.7%) and South Banat with one case (2.3%). A total of 16 (37.2%) cases were university graduates.

**Figure 3:**
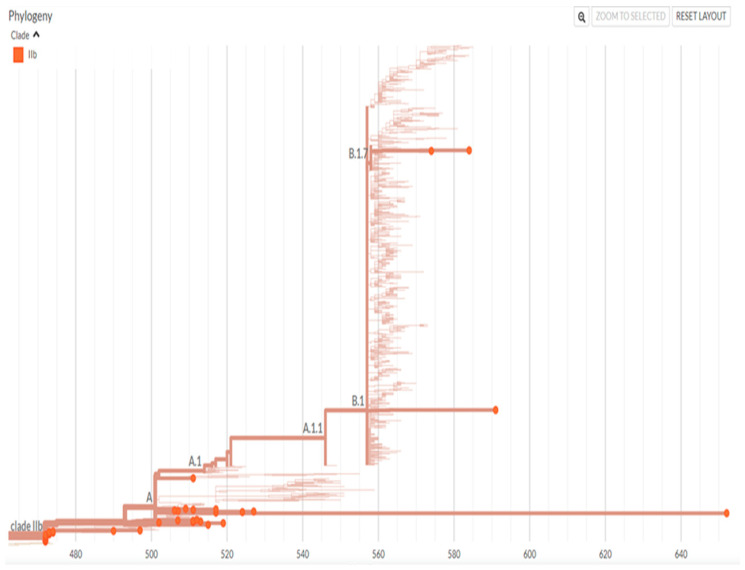
A phylogenetic tree of MPXV isolated from biological samples of mpox cases in Serbia, 2022.

Out of the total, Sanger sequencing results for 40 cases were obtained with sufficient samples for testing. These all belonged to clade IIb, classified as lineage A (37), B.1 (2), and B.1.7 (1). Figure 3 demonstrates a phylogenetic tree resulting from the Sanger sequencing of clinical specimens of mpox cases included in the study. Skin clinical specimens showed higher positivity (98.6%) than the oropharyngeal swabs (78.9%).

Out of the total, 15 cases (34.9%) were PLHIV. HIV coinfection was more often reported among cases aged 30–39 compared to those aged 20–29 and 40–59 years (*p* = 0.023). PLHIV individuals had a higher education level than the other cases (*p* = 0.030). Three cases (7.0%) were coinfected with syphilis, and one (2.3%) with the hepatitis B virus (HBV). Out of 43, 42 cases were unvaccinated against mpox, while one was vaccinated against smallpox.

As the most likely route of transmission, 32 (74.4%) cases were reported to be sexual or close physical contact. Eight cases (18.6%) declared contact with probable or confirmed mpox cases in the last month, with the time between the mentioned encounters varying between 2 and 22 days. In the past 21 days, 17 (39.5%) cases reported recent travel outside their place of residency, 5/17 (29.4%) to Spain and 3/17 (17.6%) to Greece (Table 1). Among the cases that reported traveling within the incubation period, 64.7% were from Belgrade City compared to cases residing in other NUTS-3 units (*p* = 0.039).

According to their sexual behavior, 31 (72.1%) cases were MSM, and they more often reported sexual intercourse either with multiple or unknown sexual partners compared to the others, 11/43 (27.9%) (*p* < 0.0001). In total, sexual intercourse with multiple sexual partners was reported by 14 (32.6%) individuals, and more often in those who traveled outside their place of residence (*p* = 0.02). We did not find a statistically significant difference in sexual behavior between PLHIV and the HIV-negative cases.

The overall incidence rate in Serbia was 0.64 per 100,000 population. The incidence rate was highest in the Belgrade City NUTS-3 unit (2.02 per 100,000 inhabitants). People living in Belgrade City NUTS-3 were six times more likely to develop mpox (IRR = 6.13 95% CI 1.47–25.53, *p* = 0.004) than residents of South Backa NUTS-3 (Table 2).

The highest incidence rate of 2.6 cases per 100,000 population was revealed among the cases aged 30–39. In the cases of those 30–39 years old, the presence of MPXV was diagnosed 4.5 times more often compared with the age group of 40–59 years (IRR= 4.75, 2.25–10.03, *p* < 0.0001) (Table 2).

## 4. Discussion

After the occurrence of the first registered outbreaks in Europe, the Serbian Public Health authorities decided to design and run an mpox surveillance system to identify and verify cases early and to control the spread of disease in the community. The experience of countries that faced a higher number of cases in the early stage of the mpox global outbreak around the world was helpful for Serbia to focus on previously assessed risk factors and behaviors leading to mpox transmission among particular population subgroups [20].

Our findings are consistent with previously published data related to the sex distribution of cases since, in Serbia, mpox has occurred only among males, especially MSM. Authors from Slovenia described 49 laboratory-confirmed mpox cases, mainly affecting males with a mean age of 38 [20]. The study on almost 30,000 cases from France revealed that 96.3% of cases were men with a mean age of 37 years [21].

The highest number of cases and the highest incidence rate of confirmed cases was in the Belgrade City NUTS-3 unit. Citizens from Belgrade City NUTS-3 were six times more likely to become mpox cases compared to the reference group. These findings are in line with studies conducted in the USA, where 95.7% of male cases were reported in urban areas [22,23]. The outbreak description from Peru also revealed that most of the cases were reported in urban settings, such as Lima, where the mpox burden was 76.9% [24]. These data could suggest the importance of personal behaviors, cultural barriers, stigma, and their impact on access to healthcare, testing, and vaccination, which could be reflected in the epidemiological situation in different settings, urban and rural.

The highest number of mpox cases in Serbia was notified between 26 and 31 calendar weeks, which might reflect the tourism season.

Out of the total Serbian cases, 11.6% communicated traveling to Spain 21 days before the onset of symptoms, and 7.0% to Greece. Regional travel within non-EU countries (Serbia, Bosnia and Herzegovina, and Montenegro) was reported in 9.3% of cases. Such visits might be related to mass gatherings of individuals with risk factors, such as Gay Pride or other events [25], potentially explaining the high affection of males, particularly MSM, due to practicing risky sexual behaviors, social habits, and the particular environments that they are exposed to including unknown/anonymous sexual partners, ‘sexual networking’, and attending and participating in events involving sexual activities, especially during the summer season [26].

In our study, each third case was coinfected with HIV, which is similar to other studies in European Union countries and worldwide [27,28,29,30]. A Spanish multicentric, prospective, observational study revealed that a prevalence of mpox/HIV coinfection was present in 40.0% of cases [27]. Similar findings were reported in the study from the Netherlands in the population of MSM mpox-positive cases, where the proportion of HIV coinfection was 40.0% [28]. A systematic review performed by Saavedra et al. [29] supports the fact that mpox/HIV confection varies from one-third to one-half of the total number of cases globally. Considering such conditions, it is necessary to set up prevention strategies directed toward higher-risk population groups in Serbia.

The majority of Serbian mpox cases presented with rash and fever. Regarding the topographic distribution of skin lesions, the majority of cases had a rash in the anogenital area, each third in the region of the face, head, and neck, and one quarter of them in the trunk. The rash mostly presented as pustular. Clinical characteristics of Serbian cases are in line with those that have been seen in other studies. Spanish authors found that the genital region was affected in 55.0% of cases with rash and a high proportion of trunk and skin lesions of body limbs in 57.0% [27]. The anogenital region was also highly affected among the Brazilian investigated cohort of mpox cases, where genital lesions occurred in 47.9% of cases and anal in each fifth [30]. The findings that the anogenital location was more often affected compared to other examined skin regions and the history of sexual exposure are evidence in favor that close contact was the main mode of transmission in Serbian mpox cases.

Despite the robust number of studies aimed to identify the relationship between HIV status and clinical outcomes of mpox infection (localization of the skin lesions, fever, lymphadenopathy, headache, myalgia, back pain, and asthenia), we did not find any statistical difference between the clinical characteristics in these two groups. A systematic review and meta-analysis conducted by Chinese authors revealed that it is possible to verify differences in mpox disease among cases by taking their HIV status into account in a larger number of them, so the odds ratios of the outcomes were significant for rash, cough, diarrhea, and proctitis. Additionally, the study confirmed the association of syphilis with PLHIV cases [31]. The potential explanation for the high prevalence of HIV infection among mpox cases is the risky sexual behavior associated with MSM and HIV-positive persons [32]. In our study, we did not observe higher frequencies of risky practices among PLHIV such as contact with probable or confirmed cases or sexual intercourse with multiple or unknown sexual partners.

Information collection from cases across national NUTS-3 units through enhanced national mpox surveillance is a strength of the current study. As the asymptomatic and pre-symptomatic patients were not tested, they might have consequently been missed and directed us to overestimate the onset of disease symptoms and underrecognize a certain number of them. Despite this limitation, our study underlines the importance of further research and in-depth analysis of the epidemiological characteristics of mpox.

## 5. Conclusions

In Serbia, mpox affected unvaccinated men, mostly MSM aged 30–39, living in urban areas such as Belgrade City NUTS-3. The prevalence of HIV infection among these cases is an additional public health issue, particularly among subpopulations practicing sex with multiple or unknown sexual partners. There is a need for further investigations and in-depth analyses to reveal all of the possible spread patterns of MPXV in the Serbian population and the identification of risk factors and behaviors leading to disease. Additional studies regarding knowledge, attitudes, risk perception, and practices among risk groups and the general population are required. Vaccine implementation and health promotion, emphasizing risk groups, should be the main pillars of disease prevention and control strategies in the coming future.

## Figures and Tables

**Table 1:** Socio-demographic, epidemiological, and clinical characteristics of mpox cases, Serbia, 2022.

Characteristics	*n*	%
Sex		
Male	43	100.0
Age groups		
20–29	11	25.6
30–39	22	51.2
40–59	10	23.2
Place of residence (NUTS level 3)		
Belgrade City	34	79.1
South Backa	2	4.7
Macva	2	4.7
Nisava	2	4.7
Srem	2	4.7
South Banat	1	2.2
Education level		
High school graduate	16	37.2
University graduate	16	37.2
University student	7	16.3
Undeclared	4	9.3
HIV status		
Positive	15	34.9
Negative	22	51.1
Unknown/not declared	6	14.0
Travel history 21 days prior to symptoms onset		
Yes	17	39.5
No	26	60.5
Sexual behavior		
MSM	31	72.1
Other	12	27.9
Possible route of transmission		
Sexual contact/Close physical contact	32	74.4
Unknown	11	25.6
Clinical symptoms		
Rash	43	100.0
Fever	37	86.0
Lymphadenopathy	19	44.2
Headache	16	37.2
Backpain	15	34.9
Myalgia	15	34.9
Astenia	1	2.3
Localization of skin lesions		
Anogenital	25	58.1
Face, head and neck	12	27.9
Trunk (thorax and abdomen)	11	25.6
Upper limb	8	18.6
Lower limb	4	9.3

**Table 2:** Incidence rates of mpox cases by age groups and NUTS-3, Serbia, 2022.

Characteristics	*n*	IR	IRR	95% CI	*p* Value
Age groups (in years)					
20–29	11	1.54	2.86	1.22–6.74	0.012
30–39	22	2.56	4.75	2.25–10.03	<0.001
40–59	10	0.54	1	Ref.	
Place of residence (NUTS-3)					
Belgrade City	34	2.02	6.13	1.47–25.53	0.004
Macva	2	0.75	2.28	0.32–16.19	0.396
Srem	2	0.71	2.14	0.30–15.24	0.434
Nisava	2	0.58	1.76	0.25–12.51	0.566
South Banat	2	0.38	1.16	0.11–12.84	0.901
South Backa	1	0.33	1	Ref.	

IR—incidence rate; IRR—incidence rate ratio; CI—confidence interval.

## Data Availability

Original data are available on request from the National Public Health Institute of Serbia “Dr Milan Jovanović Batut”.
